# Effect of IL-36γ expression on chronic periodontitis with an increase in NF-KB signaling pathway

**DOI:** 10.15171/japid.2019.005

**Published:** 2019-08-31

**Authors:** Amir Reza Babaloo, Adileh Shirmohammadi, Shima Ghasemi, Siamak Sandoghchian, Sahel Parvan, Nazanin Fathi

**Affiliations:** ^1^Department of Periodontics, Faculty of Dentistry, Tabriz University of Medical Sciences, Tabriz, Iran; ^2^Department of Prosthodontics, Faculty of Dentistry, Tabriz University of Medical Sciences, Tabriz, Iran; ^3^Department of Immunology, Faculty of Medicine, Tabriz University of Medical Sciences, Tabriz, Iran; ^4^Dentist in Private Practice, Tabriz, Iran; ^5^Faculty of Dentistry, Tabriz University of Medical Sciences, Tabriz, Iran

**Keywords:** Chronic periodontitis, Inflammation, TLR4, IL-36y, NF-KB

## Abstract

**Background:**

An inappropriate inflammatory response is the cause of many common diseases, especially periodontitis. Considering that no studies have been carried out to investigate the effect of IL-36γ on chronic periodontitis, this study aimed to investigate the inflammatory mechanism of IL-36γ by stimulating macrophage cells using NF-KB pathway.

**Methods:**

This experimental study was performed on 50 healthy individuals and 50 subjects with chronic periodontitis. In this study, macrophage cells were extracted first, and then RNA was isolated from all the samples using TRIzol method. Subsequently, the rate of IL-36γ gene expression was analyzed and compared using real-time PCR technique. Additionally, immunofluorescence (IF) technique was used to investigate the rate of inflammation. The rate of NF-Kb expression was also measured via western blot technique. Finally, statistical analysis of the samples was carried out using appropriate statistical methods with SPSS 17.

**Results:**

The results showed that the rate of IL-36γ expression in subjects with periodontitis was higher compared to healthy subjects (P<0.05). Moreover, the results showed that following treatment of cells with TLR4, the rate of IL36γ expression increased significantly, especially during the 12-hour period after treatment.

**Conclusion:**

This indicates that after stimulating the TLR pathways, the rate of IL-36γ expression will probably increase.

## Introduction

Periodontal pathogens are necessary for the onset of periodontal diseases. However, the prevalence of the disease and the severity of tissue destruction highly depend on the nature of host‒microbe interactions. Host response to periodontal infections needs the expression of some bioactive factors such as proinflammatory and anti-inflammatory cytokines, growth factors and enzymes due to the activation of multiple signaling pathways. In fact, most of the tissue, destruction is due to an increase in and inactivation of mediators related to inflammation and destructive subgingival bacterial plaque enzymes.^1^

Inflammation includes some biochemical and cellular changes. Inappropriate inflammatory response is the cause of many common diseases such as periodontitis. Inflammatory cells are activated by cytokines, bioactive molecules, and MAMPs in the area and regulate the activity of other cells by producing other inflammatory mediators and affecting the hemostasis of mineral and non-mineral tissues in the periodontium. The cytokines responsible for the initial response to microbial attack include IL-6, IL-1β, IL-1α and TNF-α. The members of interleukin-1 family as the effective molecules in the innate immune system are the vital regulators of various inflammatory disorders and all immune cells express or are under the influence of the members of IL-1 family.^2,3^

IL-36γ regulates IL-1β levels through NF-Kb signaling pathway and the factors are regulated by MAPK such as c-jun by binding to IL-36R receptor. In addition, IL-36γ activates MAPK and NF-kB signaling pathways in epithelial cells.^4,5^ TLRs exist on the cells of innate immune system (cells involved in the first line of defense), including neutrophils, monocytes/macrophages, and epithelial cells. Such cells, playing a significant role in detecting and killing microorganisms, indicate different TLRs which cause different immune responses to a specific pathogen.^6,7^ Studies indicated that IL-36γ works as a proinflammatory cytokine in inflammation.^8^ Such cytokines are able to stimulate the performance of Th17 by inducing the relevant cytokines.^9^ A study showed that macrophages and dendritic cells derived from human monocytes that expression of Th-17 chemokine could create inflammatory responses via IL-36γ. In addition, the lipopolysaccharide (LPS) derived from *E. coli* and *P. gingivalis* significantly expresses IL-36γ, which is different for IL-36α and IL-36β. As it was mentioned, these factors affect the role of IL-36γ in inflammations while its role in the inflammation of chronic periodontitis has not been studied yet.^10,11^

Studies on IL-36γ indicated that TLR4 can increase IL-36γ expression and can activate the signaling pathway of nuclear factor – KB (NF-KB) and MAPK (mitogen activated protein kinase).^12^ Since no study has been conducted on the effect of IL-36γ with the assessment of the effect of NF-KB factor on chronic condition of periodontitis, the present study investigated the effect of IL-36γ expression on chronic periodontitis through an increase in NF-KB signaling pathway.

## Methods

In this study, all the laboratory stages were conducted in the Immunology Laboratory of Immunology Research Center, Tabriz University of Medical Sciences. First the gingival tissues derived from a sub-marginal flap were prepared and then sent to the Immunology Laboratory in normal saline solution. Then, the macrophages were isolated from the tissue samples using the MACS (magnetic activated cell sorting) technique. In this way, the cells were isolated by specific nanoparticles which contain specific antibodies of CD14 and CD16. After the isolation, the cells were cultured in 1640 RPMI culture medium with 1% erythromycin.

### Evaluation of Gene Expression

First, the cells were stimulated by TLR4 proteins at 2-, 4-, 8-, 12- and 24-hour intervals. Then, the cell mRNA was extracted by TRIzol technique and IL-36γ level in macrophages was evaluated in a real-time PCR device (Rotogen Company). In this method, β-actin gene was selected as control. In addition, some part of the tissue was measured directly with IL-36γ and β-actin primers through real-time PCR technique.

### Immunofluorescence Technique

In order to prove inflammation, the samples were placed in a blocking buffer for one hour after paraffinization of the cells. The primary antibodies anti-CD4 and anti-IL-17 were placed at room temperature for two hours. After the rinse, the secondary antibody was added for one hour. Then, 50 μL of hochest was poured onto the slides after incubation and studied under an immunofluorescence microscope.

### Western Blot Technique

The tissues were electrophoresed on 12% SDS Page gel and then transferred to PVDF transfer plates. The plates were blocked with 5% skimmed milk with 1% Tween in PBS for one hour at room temperature and then incubated for one night by anti-TLR4 and anti-NF-KB purchased from Abcam Company. The effect was measured by Amercontrol software.

### The Design of Primers by a Computer

In order to facilitate the primer design process and minimize the required time, a computer software program such as Oligo 7 can be used to design and blast the primers. Many types of computer software programs are used to search the sequence of an appropriate primer region and select the best primer pairs with favorable features.

### Real-time PCR

All real-time PCR reactions were performed on (Rotor Gene TM 6000 (Corbett) device. The relevant reactions were performed in three steps. The standard curve was drawn based on the logarithm of the horizontal axis of cDNA concentration and vertical axis of threshold cycles for each gene. The PCR yield was determined for each gene based on the standard curve. From the non-cDNA sample, ‘no template control’ (NTC) was used as a negative control. An amplification curve was drawn for each reaction. The analysis was conducted according to the comparison of the threshold cycles of patients having chronic periodontitis to healthy samples.

The research results were reported using descriptive statistics (mean ± standard deviation and percentage) to compare the level of IL-36γ expression. ANOVA was used to compare this amount in two pathways at different times. Statistical analysis was conducted using SPSS 17 and the significance level was defined at P<0.05.

## Results

This study was experimental and 100 subjects were included in the study, of which 50 subjects were in the healthy group, with 50 in the patient group.

**Figure 1:**
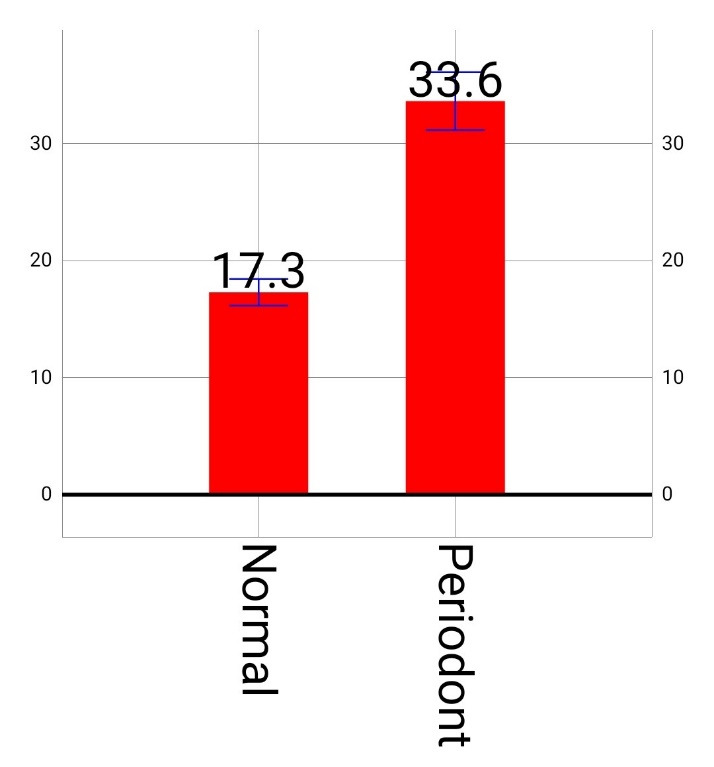
Comparison of gene expression between the two groups.

As can be observed in Figure 1, the percentage of expression was 17.3% in the healthy group, with 33.6% in the patient group. The results indicated that that expression of IL-36γ in subjects with periodontitis was significantly different from the healthy subjects (P<0.001). This significant difference was due to the presence of inflammation in the subjects with periodontitis (Figure 1).

**Figure 2:**
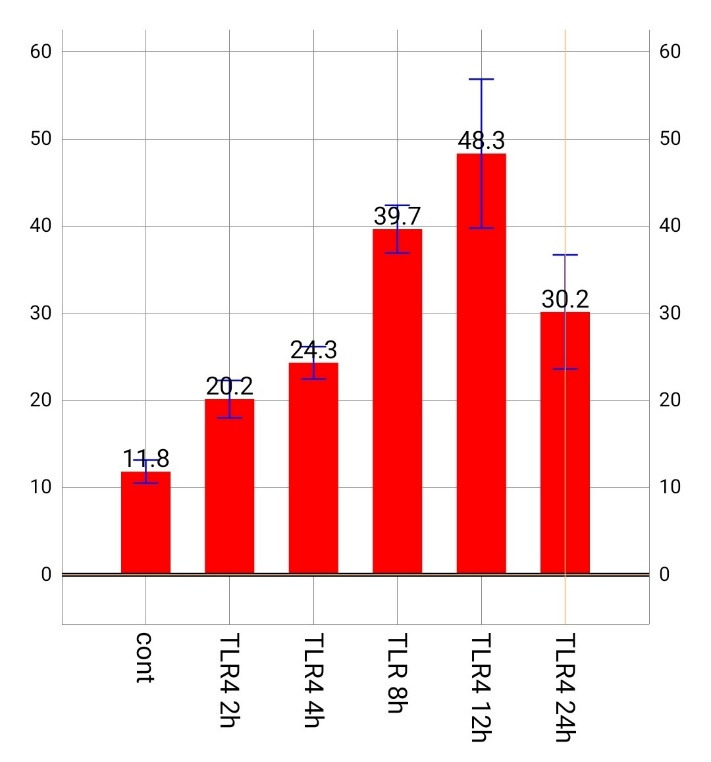
Comparison of TLR4-treated gene expression at different time intervals.

Comparison of gene expression in TLR4-treated cells was investigated at 2, 4, 8, 12 and 24 hours. The results showed that IL-36γ gene expression increased with an increase in the duration of treatment with TLR4 and this increase peaked 12 hours later (48.3%). This amount decreased to 30.2% after 24 hours due to its reduced effectiveness. Such a difference in gene expression was reported significant at different times according to the results of a repeated measurement test (P<0.001) (Figure 2).

**Figure 3:**
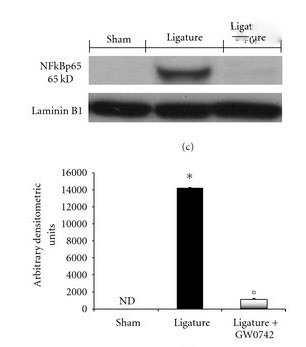
Comparison of Nf-KB gene expression to the sham group.

In this study, the amount of Nf-KB gene expression in patients with periodontitis was evaluated in IL-36γ-carrying cells. As shown in Figure 3, Nf-KB in the periodontitis group increased in comparison to the sham group. In other words, IL-36γ can increase transcription by enhancing TLR4 pathway through MYD88 proteins as well as increasing the JAK and STAT pathways. Nf-KB levels increased with an increase in inflammatory cytokines (Figure 3).

## Discussion

Chronic periodontitis is one of the infectious diseases caused by inflammation in the supporting tissues of teeth, leading to the loss of attachments and the alveolar bone.^13^ Many studies have considered the role of inflammatory and immune responses, especially humoral immune responses, in the pathogenesis of this disease in recent years. These studies indicated that factors such as the complement system, collagenase and inflammatory cytokines, including the gene family of IL-1, TNF-α and IL-6, as important factors involved in the inflammatory system, are of great importance.^14-16^

In particular, IL-36γ is one of the strongest inflammatory cytokines playing a main role in the reaction of host to gingivitis. Interestingly, the expression of IL-36γ affects other cytokines of the IL-36 family. The cause of this effect is still unknown as how the effects of all the three various types of IL-36 occur through an IL-36R receptor. However, it might have different kinds of bioavailability in vivo, which might result in different inflammatory responses with different severities and durations.^5^

The results of this study revealed that IL-36γ expression in the periodontitis group was significantly higher in comparison to the healthy group. In a study by Kurşunlu et al^17^ on the levels of IL-36γ expression in periodontal patients, the results indicated no significant differences between different groups of invasive and chronic periodontitis and healthy subjects. However, IL-36γ values in the chronic periodontitis group were higher than other groups, which is different from the results of this study. However, the values for IL-36β in invasive periodontitis samples were more than the other samples and there was a statistically significant difference. The results of this study demonstrated that cytokines such as IL-36γ, in contrast to the family of IL-1 cytokines (the role of these proteins was mentioned in the progression of periodontal disease in previous studies) have no role in the pathogenesis of periodontal disease.^18^ Such results indicated that IL-36γ cannot activate signaling functions related to IL-1 and likely act through alternative signaling pathways.^19^ In addition, the results of other studies showed that the production of these new molecules by inflammatory cells is low.^5,20^ The results of a study by Huynh et al^21^ indicated that IL-36γ applies inflammatory responses through dendritic cells derived from monocytes as well as macrophages that includes the expression of neutrophil chemokines such as IL-8, CXCVL1, IL-17 and CCL20. IL-36γ is similarly stimulated in the oral epithelial cells. IL-17 not only stimulates the expression of significant regulators which attract neutrophils but also leads to IL-36γ expression in epithelial cells.

In addition, the results related to gene expression in TLR4-treated cells in the present study indicated that IL-36γ gene expression increased with an increase in the duration of treatment by TLR4 and such an increase peaked 12 hours later. However, this amount decreased after 24 hours. The results of a study by Sara et al^22^ revealed that TLRs expression in chronic periodontitis significantly increased compared to healthy and gingivitis patients and TLR4 expression increased more than TLR2.^22^ Another study by Yamaguchi et al^23^ investigated the effect of gingival plaque on the production of inflammatory cytokines by inducing TLR2 and TLR4 in peripheral blood mononuclear cells. The results indicated that levels of inflammatory cytokines, including TNFα, IL-6 and IL-8, were related to the ability of plaque to induce TLR4 pathway. Furthermore, it was found that cytokine production was inhibited by using TLR4 antagonists.

It has been recently found that IRF6 (IFN regulatory factor) is the key regulator for the expression of IL-36γ through oral epithelial cells in response to gingivitis. IRF6 plays a critical role in regulating inflammatory cytokines caused by TLR2. Oral epithelium cells as the first cells encountering gingivitis play a significant role in oral periodontium.^24^ The results of some studies indicated that IRF6 stimulates the expression of IL-1 family of inflammatory cytokines like IL-36γ when oral epithelial cells encounter gingivitis.^21^

## Conclusion

Based on the results, expression of IL-36γ increased in subjects with periodontitis compared to healthy subjects. In addition, it was found that the level of IL-36γ expression significantly increased after treating the cells with TLR4. The maximum amount of it was related to 12 hours after the treatment, indicating that the expression of IL36γ increased by stimulating TLR pathways.

## Authors’ Contributions

ARB and ASh carried out surgeries and prepared the samples. SS was responsible for laboratory procedures. SP and SG were responsible for case selection, screening, coordination and preparation of the proposal; SP and NF prepared the manuscript.

## Competing Interests

The authors declare no competing interests with regards to the authorship and/or publication of this article.

## Ethics Approval

This study was approved by the Ethics Committee of Tabriz University of Medical Sciences under the code IR.TBZMED.REC.1397.152
